# The Psychometric Parameters of the Farsi Form of the Arabic Scale of Death Anxiety

**DOI:** 10.1155/2017/7468217

**Published:** 2017-06-18

**Authors:** Mahboubeh Dadfar, Ahmed M. Abdel-Khalek, David Lester, Mohammad Kazem Atef Vahid

**Affiliations:** ^1^School of Behavioral Sciences and Mental Health-Tehran Institute of Psychiatry, Iran University of Medical Sciences, International Campus, Tehran, Iran; ^2^Department of Psychology, Faculty of Arts, University of Alexandria, Alexandria, Egypt; ^3^Psychology Program, Stockton University, Galloway, NJ, USA; ^4^Department of Clinical Psychology and Department of Health Psychology, School of Behavioral Sciences and Mental Health-Tehran Institute of Psychiatry, Iran University of Medical Sciences, Tehran, Iran

## Abstract

The aim of this study was to describe the psychometric properties of the Farsi Form of the Arabic Scale of Death Anxiety (ASDA). The original scale was first translated into Farsi by language experts using the back translation procedure and then administered to a total of 252 Iranian college students and 52 psychiatric outpatients from psychiatric and psychological clinics. The one-week test-retest reliability of the Farsi version in a sample of college students was 0.78, indicating good temporal stability and corroborating the trait-like nature of scores. Cronbach's *α* was 0.90 for the college students and 0.92 for the psychiatric outpatients, indicating high internal consistency. Scale scores correlated 0.46 with Death Obsession Scale scores, 0.56 with Death Depression Scale scores, 0.41 with Death Anxiety Scale scores, and 0.40 with Wish to be Dead Scale scores, indicating good construct and criterion-related validity. A principal component analysis with a Varimax rotation yielded four factors in the sample of Iranian college students, indicating a lack of homogeneity in the content of the scale. Male students obtained a significant higher mean score than did females. It was concluded that the Farsi ASDA had good internal consistency, temporal stability, criterion-related validity, and a factor structure reflecting important features of death anxiety. In general, the Farsi ASDA could be recommended for use in research on death anxiety among Iranian college students and psychiatric outpatients.

## 1. Introduction

The understanding of death was one of the most important issues in ancient times and remains a persistent question facing humans today. Death anxiety is a negative and apprehensive feeling that one has when thinking about death and dying. Death anxiety is used interchangeably with fear of death [1–5].

Cross-cultural studies are important ways of establishing the parameters of any particular phenomenon including death anxiety [6]. For example, emerging research on non-Western religions suggest that they may be associated with distinctive forms of death anxiety, such as the intense apprehension reported by many Muslims regarding “the torture of the grave” (a special and horrific set of punishments that can be applied to the bodies of the dead) [7], and so there is a need for culturally attuned research [8]. In recent years, many cross-cultural comparisons have been carried out on death anxiety [9–12].

There are several tools available for the assessment of attitude toward death and dying such as the Death Concern Scale (DCS), the Collett-Lester Fear of Death Scale (CLFDS), Templer's Death Anxiety Scale (DAS), the Arabic Scale of Death Anxiety (ASDA), the Reasons for Death Fear Scale (RDFS), the Death Obsession Scale (DOS), the Death Depression Scale (DDS), the Wish to be Dead Scale (WDS), the Multidimensional Fear of Death Scale (MFODS), the Death Attitude Profile-Revised (DAP-R), the Multidimensional Orientation toward Dying and Death Inventory (MODDI-F), and other scales [13–30]. The field of death anxiety contains both uni- and multidimensional scales. The most famous anxiety used scale (DAS by Templer) is a unidimensional scale.

The Arabic Scale of Death Anxiety (ASDA) is the focus of the present research because there is a common element in Arabs and Iranians, namely, the religion of Islam, as well as sharing a cultural context and a number of historical events. The ASDA was developed and validated by Abdel-Khalek (2004) in a sample of undergraduates in three Arab countries, Egypt, Kuwait, and Syria [7]. The ASDA was developed originally in Arabic but now has English, Spanish, Turkish, and Chinese versions. Many studies have been carried out using the ASDA [31, 32]. Abdel-Khalek and Lester (2009) reported that Kuwaiti undergraduate students had a significantly higher mean score on the ASDA than did their American counterparts. Scores on somatic symptoms inventory were positively correlated with death anxiety scores, indicating that students who enjoyed good physical health are less concerned with death [2].

Abdel-Khalek et al. (2009) examined the sex and national differences in seven Arab and Western countries: Egypt, Kuwait, Lebanon, Syria, Spain, the United Kingdom, and the United States. The results showed that sex-related differences on the ASDA were statistically significant in all countries except the United Kingdom, with women having a higher mean score than did men. All the Arab samples, except the Lebanese men, had significantly higher mean scores on the ASDA than did their Western counterparts. The authors explained these differences in the light of higher emotionally responsiveness in the Arab samples, differences in individualism, collectivism, and secularism, and the lower per capita income in the Arab countries [9].

Thabet et al. (2013) showed that on the ASDA 59.5% of a sample of Palestinian adults had fears of the punishment in their grave, 51.9% had fears of having cancer, and 50.5% were worried that death will take someone they love. The least common items to be feared were the following: “walking in a cemetery frightening them” (11%), “upset when seeing a funeral” (10.4%), and “afraid to visit a cemetery” (9.1%). Palestinian women obtained a statistically significant higher mean ASDA score than did men. The total ASDA scores were positively associated with the following trauma items: forced to leave your home during the war, destruction of your personal belongings during incursions, hearing the sounds of the jetfighters, deprivation of toilet facilities, and leaving the room at home when detained. The ASDA was negatively associated with witnessing the killing of a close relative [33].

Clear and compelling reasons exist for the translation of the ASDA into the Farsi language and the study of its psychometric properties in order to investigate the cultural, ethnic, and sociodemographic factors that can influence the intensity of death anxiety in Iran. Despite the good psychometric characteristics of the ASDA and its applicability in British, USA, Spanish, Turkish, and Chinese samples and several Arab countries, there are no published studies on Iranian college students and psychiatric outpatients. The Arab countries and Iran share religion of Islam but they are different in language. Therefore, our research was done to adapt and implement the ASDA in two samples of Iranian college students and psychiatric outpatients. The ASDA would be useful for research into personality, in clinical practice, and in cross-cultural comparisons.

The aims of the present study were (a) to develop a Farsi version of the ASDA, (b) to evaluate its psychometric characteristics, that is, its reliability, validity, and factorial structure in Iranian college students and psychiatric outpatients, (c) to explore associations between death anxiety and other psychological variables, and (d) to compare the mean ASDA scores in Arabic and Western samples (Egypt, Kuwait, Lebanon, Syria, Spain, the United Kingdom, and the United States) and an Iranian sample.

## 2. Method

### 2.1. Participants

Three samples were recruited: a convenience sample of 252 Iranian volunteer college students, another sample of 58 college students to explore the construct validity of the ASDA, and 52 psychiatric outpatients. They were selected from the colleges at the Iran University of Medical Sciences, and psychiatric and psychological clinics at the School of Behavioral Sciences and Mental Health-Tehran Institute of Psychiatry at the Iran University of Medical Sciences. Informed consent was obtained and anonymity was guaranteed.

The mean age of students was 25.8 years (SD = 5.3); 47.9% were males; 71.5% were studying to be general physicians (GP); 13.1% were studying clinical psychology, 1.1% mental health, and 8.6% other. They responded to the scales and questions in individual sessions.

The mean age of psychiatric outpatients was 33.0 years (SD = 8.4). The mean duration of their mental disorder was 3.5 years (SD = 3.3). Of this sample, 65.4% were females, 57.7% were single, and 40.4% were married. In this group, 17.3% had a depressive disorder, 11.5% an anxiety disorder, 3.8% mixed diagnosis, and 1.9% other disorders. There were missing data for 65.5% of diagnoses. They responded to the scales in individual sessions.

### 2.2. Measures

The Arabic Scale of Death Anxiety (ASDA) consists of 20 statements. Each item is answered on a 5-point intensity scale anchored by 1 (No) and 5 (Very much). In its Arabic form, the alpha reliabilities ranged from 0.88 to 0.93, and the item-remainder correlations ranged between 0.27 and 0.74 [7]. The one-week test-retest reliability was 0.90, indicating good internal consistency and temporal stability. The total score can range from 20 to 100, and a high score indicates high death anxiety. The correlations between the ASDA and the Templer's Death Anxiety Scale (DAS) ranged between 0.60 and 0.74, indicating high convergent validity. Pearson correlations between the total scores on the DAS, the ASDA, and the Collett-Lester Fear of Death Scale (CLFDS) were significant and positive. One high-loaded factor was extracted from the aforementioned three scales and labeled General Death Anxiety, indicating good convergent validity of the scales [7]. In study of Sarıçiçek Aydoğan et al. [34], the Cronbach's alpha coefficient for Turkish version of the ASDA was 0.86, and the ASDA scores were highly correlated with DAS scores (*r* = 0.68, *p* < 0.001).

In the present study, first, the Arabic version of the ASDA was translated into the Persian language by bilingual four native, Iranian, Persian-speaking individuals. Second, this version was back-translated into Arabic by another person who was a native Arabic speaker (a Lebanese general physician). Finally, the Farsi version of the ASDA was compared to its English version, and differences in the three versions were resolved. In order to study the construct validity of the ASDA, 58 college students completed the Farsi versions of the following five scales.

*(1) Death Obsession Scale (DOS) [13]*. The DOS has 15 items and is answered on a five-point rating scale: 1: No, 2: A little, 3: A fair amount, 4: Much, and 5: Very much. Total scores can range from 15 to 75. A typical item is “I am preoccupied by thoughts of death.” Cronbach's alpha was 0.90. There was significant correlation between DOS and ASDA scores in their Arabic forms [7]. The Pearson correlation between the DOS and the Death Concern Scale scores was 0.45 for Iranian nurses [5]. 

*(2) The Death Depression Scale (DDS) [35]*. The DDS has 17 items, and two different formats (a true/false or yes/no format and a five-point Likert format). In the false-true format, items are answered (0) false and (1) true, and the true/false format was used for the present study. The DDS has two items to control an acquiescence response set (items 11 and 12). Total scores can range from 0 to 17. A typical item is “I get depressed when I think about death.” Cronbach's alpha for the Farsi DDS was 0.76 [36] and 0.84 [37]. 

*(3) Templer's Death Anxiety Scale (DAS) [30]*. The DAS has 15 items answered with a true or false response. The DAS score can range from 0 (lack of death anxiety) to 15 (very high death anxiety). A typical item is “I am very much afraid to die.” The scores on the DAS are significantly associated with the subscales of the Collett-Lester Fear of Death Scale [26]. 

*(4) The Wish to Be Dead Scale (WDS) [27]*. The WDS has 10 items and can be used with two possible formats: a true/false format and a Likert-type response format. The true/false format was used for the present study and is scored true (1) and false (0). A typical item is “I occasionally day-dream about being dead.” Cronbach's alpha was 0.82, and the two-week test-retest correlation was 0.87 [27]. Cronbach's alpha for the Farsi version of the WDS was 0.73 [38] and 0.82 [39]. 

*(5) The Love of Life Scale (LLS) [40]*. The LLS is a 16-item scale to assess the respondent's love of life, answered on a 5-point scale: No (1); A little (2); Moderate (3); Much (4); and Very much (5). A typical item is “Life is full of pleasures.” The total score can range from 15 to 75, and the high score indicates a strong love of life [40]. Cronbach's alpha for the Persian version of the LLS was 0.94 [41].

## 3. Results

### 3.1. Factor Analysis of the ASDA

The criteria for the factor analysis were evaluated using the Kaiser-Meyer-Olkin Measure of Sampling Adequacy (KMO) and the Bartlett Test of Sphericity. The KMO was 0.889, indicating the adequacy of the sample of college students, and the Bartlett's Test of Sphericity was 2.529*E*3 (df = 190, *p* < 0.001) indicating that the factor analysis was justified in the college student sample. As for the sample of mental patients, the KMO was 0.834, indicating the adequacy of sampling, and the Bartlett's Test of Sphericity was (640.932, df = 50, *p* < 0.001) indicating that the factor analysis was justified in the sample of psychiatric outpatients. To investigate the factor structure of the scale, a principal component analysis with a Varimax rotation and Kaiser Normalization were used. The salient loading was defined as ≥0.50.

**Figure 1:**
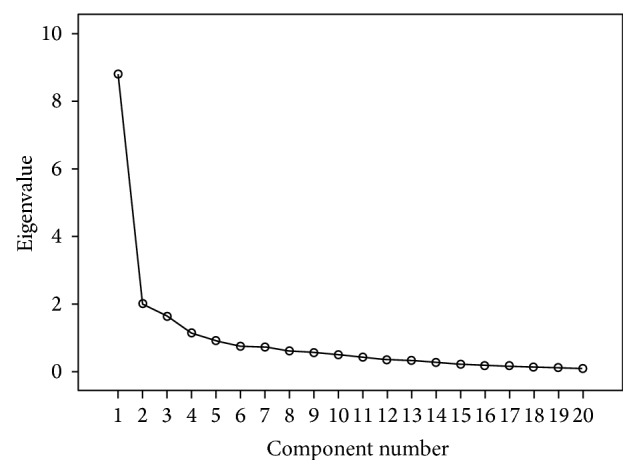
Scree Plot of the college students.

Four components with eigenvalues greater than one were retained in the sample of college students as reported in Table 1 and Figure 1. Inspection of this table reveals that Factor 1 (8 items) explained 21.59% of the observed variance and was labeled “fear of lethal disease and death.” It included the following items: “I fear death whenever I become ill,” “the possibility of having a surgical operation terrifies me,” “I am afraid of suffering heart attack,” “I fear getting a serious disease,” “I am afraid of sleeping and not waking up again,” “the pain accompanying death terrifies me,” “I am afraid of getting cancer,” and “I fear death.”

Factor 2 (4 items) explained 16.76% of the observed variance and was labeled “fear of dead people” and included the following items: “I fear looking at the dead,” “I am afraid of looking at a corpse,” “I get upset by witnessing a funeral,” and “the sight of a dying person frightens me.”

Factor 3 (3 items) explained 11.30% of the observed variance and was labeled “fear of tombs”. It included the following items: “I fear visiting graves,” “witnessing the burial procedure terrifies me,” and “I dread walking in graveyards.”

Factor 4 (3 items) explained 11.27% of the observed variance and was labeled “fear of the afterlife” and included the following items: “I am apprehensive of unknown things after death,” “I fear the torture of the grave,” and “I am preoccupied with thinking about what will happen after death."

Also correlations between the factors for men were from 0.517 to 0.826, and for women were from 0.532 to 0.767, respectively.

**Figure 2:**
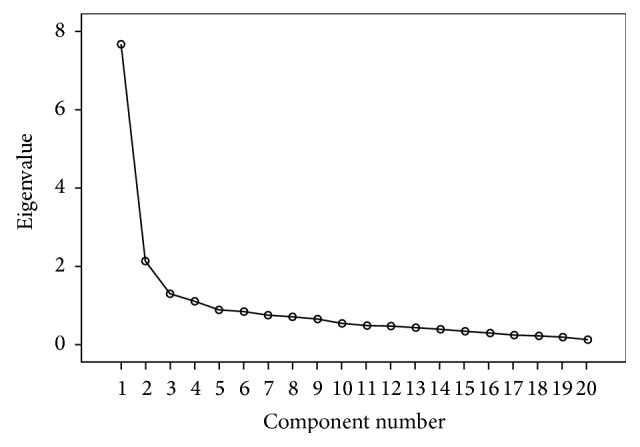
Scree Plot of the psychiatric outpatients.

Four components with eigenvalues greater than one were retained in the sample of psychiatric outpatients (see Table 2 and Figure 2). Factor 1 (8 items) explained 43.97% of the observed variance and was labeled “fear of lethal disease” and included the following items: “the possibility of having a surgical operation terrifies me,” “I am afraid of suffering heart attack,” “I fear getting a serious disease,” “Witnessing the burial procedure terrifies me,” “I get upset by witnessing a funeral,” “the sight of a dying person frightens me,” “I am afraid of getting cancer,” and “I fear death.”

Factor 2 (6 items) explained 10.05% of the observed variance and was labeled “fear of dead people and death” and included the following items: “I fear looking at the dead,” “I worry that death deprives me of someone dear to me,” “I am apprehensive of unknown things after death,” “I fear the torture of the grave,” “the sight of a dying person frightens me,” and “talking about death upsets me.”

Factor 3 (4 items) explained 8.21% of the observed variance and was labeled “fear of tombs” and included the following items: “I fear visiting graves,” “I am afraid of looking at a corpse,” “I dread walking in graveyards,” and “I am afraid of sleeping and not waking up again.”

Factor 4 (1 item) explained 5.75% of the observed variance and was labeled “fear of postmortem events” and included the item “I am preoccupied with thinking about what will happen after death.” Because this factor contains only one salient loading, we considered it as a residual factor.

### 3.2. Reliability of the ASDA

For the college student sample, Cronbach's alpha was 0.90, the split-half reliability was 0.85, the Spearman-Brown coefficient was 0.91, and the Guttman split-half coefficient was 0.91. The one-week test-retest reliability was 0.78. In the sample of psychiatric outpatients, Cronbach's alpha was 0.92, the split-half reliability was 0.84, the Spearman-Brown coefficient was 0.91, and the Guttman split-half coefficient was 0.91, indicating good to high reliability. In the sample of college students, the intercorrelations between the items ranged from 0.08 to 0.81 and the item-total correlations ranged from 0.44 to 0.75. The intercorrelations between the items ranged from 0.03 to 0.78, and the item-total correlations ranged from 0.39 to 0.84 in the sample of psychiatric outpatients. The correlation between the total score on the ASDA and age was −0.49 (*p* < 0.05) and with duration of mental disorder *r* = 0.87 (*p* < 0.01).

### 3.3. Construct Validity of the ASDA

Table 3 presents the correlations between ASDA scores and the other scales scores in the sample of college students. Inspection of this table reveals that these correlations ranged between 0.40 and 0.56 except for the correlation with LLS (ns). All the correlations were in the expected direction, thereby indicating good construct validity of the Farsi ASDA.

### 3.4. Intercultural Comparisons of the ASDA

The mean of the ASDA total score was 41.40 (SD = 12.73) in college students and 54.34 (SD = 18.85) in psychiatric outpatients. The mean of male students was 42.82 (SD = 13.99) and of female was 39.94 (SD = 11.15), a statistically significant difference (*p* < 0.001). The mean score of the male patients was 51.38 (SD = 11.18) and that of the female patients was 55.91 (SD = 16.41), a nonsignificant difference (*t* = 1.04, df = 50, n.s.). The mean score for the depressed patients was 57.77 (SD = 17.96), and for the anxiety patients was 53.66 (SD = 13.66), a nonsignificant difference.

Table 4 sets out the descriptive statistics and the “*t*” values for the Arabic, Western, and Iranian college students. The mean ASDA score of the male Iranian students was less than the mean score of the Arab students from Egypt, Kuwait, and Syria, as well as UK. The mean ASDA score of the female Iranians was lower than that for all the Arab and Western samples.

## 4. Discussion

The main aim of the present study was to explore the reliability, validity, and descriptive statistics of the Farsi version of Abdel-Khalek's (2004) Arabic Scale of Death Anxiety (ASDA). The results indicate that the ASDA has good to high internal consistency and test-retest reliability (ranging from 0.78 to 0.92) in the Iranian samples of college students and psychiatric outpatients. Different methods of assessing reliability were used to demonstrate the high reliability of the scale. We used one-week test-retest reliability, to be consistent with the original studies on the scale [7, 9] and study of Qiu et al. [42], and appropriately compare the results. Generally speaking, the majority of studies in this respect used one-week test-retest interval.

The Varimax rotation was used because the factors obtained were better differentiated, making interpretation easier. A high loading (>0.50) was also used to more clearly differentiate the factors and to retain strong, high-loaded factors. The principal components analysis identified four components of the ASDA in college students as follows: fear of lethal disease and death, fear of dead people, fear of tombs, and fear of postmortem events. Three components were identified of the ASDA in a sample of psychiatric outpatient, that is, fear of lethal disease, fear of dead people and death, and fear of tombs. Thus, the Farsi ASDA was composed of meaningful components related to death anxiety. It would be of interest in future research to explore the correlates of each component separately. These results on college students were similar to those reported by Abdel-Khalek (2004) who extracted four components in an Egyptian college students sample, labeled “fear of dead people and tombs,” “fear of postmortem events,” “fear of lethal disease,” and “death preoccupation.” The first two factors were almost completely identical in three Arab countries [7]. Sarıçiçek Aydoğan et al. (2015) obtained five factors for Turkish version of the ASDA, which accounted for 65.6% of the total variance in college students, labeled fear provoked by visual stimuli related to death; fear of physical and psychological pain related to death; fear of other situations reminding of death; fear of postmortem events; and fear from the act of dying [34]. Dadfar and Bahrami (2016) yield five factors for the ASDA, which accounted for 72.49% of the total variance in Iranian middle-aged, labeled fear of death and fear of dead people; fear of postmortem events and fear of tombs; fear of lethal disease; preoccupation with after death, and fear of death in sleep; and fear of deprivation of own loved ones [31]. Qiu et al. (2016) found three factors for Chinese version of the ASDA, which accounted for 57.09% of the total variance labeled “fear of dead people and tombs,” “fear of lethal disease,” and “fear of postmortem events” [42]. There was some similarity in specific factors between the previous results and the present findings.

A total score on the scale was used instead of the four separate scores for each of the factors because the factors identified in the various studies on the ASDA were not consistent with one another. This may be due to the different characteristics of the samples and to the capricious nature of exploratory factor analysis. Therefore, until a consistent factor structure can be identified, we decided to use the total score. Also the original author [7] used the total score in his study of three cultures. So, to compare the results, the total score was used in this study too. Further, all obtained subconstructs of the ASDA, “fear of lethal diseases and of death,” “fear of dead people,” and “fear of tombs,” are different aspects of the main concept, that is, death anxiety.

We predict that ASDA scores would be associated with scores on the other scales, and, therefore, this is construct validity. The total score on the ASDA reflects anxiety relating to death. The construct is death anxiety, that is, the anxiety people experience when they think about the possibility of dying or are reminded of the issue of death. Fear of dead people reminds them of the fact that they themselves might die, as the tombs make them think of the inevitable. The main issue is that people try to avoid thinking about their ultimate fate, but when they are confronted with objects or situations that remind them of the inevitability of their own demise, they may experience significant anxiety. Table 3 reflects the convergent and divergent validity of the scale with scales that are theoretically related to death anxiety.

Scores on the ASDA were associated with several scores of death distress (death depression, death obsession, death anxiety, and the wish to be dead). These associations provide evidence for the construct validity of the ASDA and are consistent with the original study of Abdel-Khalek (2004) on Arab samples. He reported that there were significant correlations between the ASDA and death depression, death obsession, reasons for fearing death, general anxiety, depression, obsession-compulsion, and neuroticism [7]. In the study of Qiu et al. (2016) the ASDA scores were highly significantly correlated 0.54 with long form Multidimensional Orientation toward Dying and Death Inventory (MODDI-F/chin) scores [42]. Therefore, it is safe to conclude that the Farsi version of the ASDA is useful for assessing death anxiety in clinical and nonclinical Iranian samples.

In this study, the mean ASDA total score was 41.4 in college students and 54.3 in psychiatric outpatients. The difference between the two mean scores indicated the discriminant validity of the ASDA for the present samples. Iranian male students obtained a significantly higher mean ASDA score than did their female counterparts. This result is in conflict with other studies. Abdel-Khalek (2005) found that gender differences were statistically significant on the ASDA, but females obtained a higher mean score than did males in three normal (nonclinical) samples, anxiety disorder patients, and schizophrenic patients [43]. Lester et al. (2006-2007) reported that there were strong sex differences in the DAS scores of men and women [11]. Abdel-Khalek et al. (2009) found that, on the ASDA, women obtained statistically significant higher mean scores than did their male peers in Egypt, Kuwait, Lebanon, Syria, Spain, and the United States (with the exception of the United Kingdom) [9]. Thabet et al. (2013) found that Palestinian women reported more death anxiety than did men, and they suggested that their result was because men were less willing to admit their fears openly [33]. Kastenbaum (2000) hypothesized that the greater anxiety about death in women is because they are most often the primary caretakers for those dying [44]. Dattel and Neimeyer (1990) introduced the emotional expressiveness hypothesis to explain the high mean score of women in death anxiety [45]. In sum, the higher mean ASDA score of men than that of their female counterparts in the present study was not consistent with the majority of previous studies. The reason for this finding was not clear and no explanation can be proposed.

The ASDA items receiving the highest mean scores in the present sample of college students were as follows: item 6 “I worry that death deprives me of someone dear to me” (3.01), item 19 “I am afraid of getting cancer” (2.82), and item 10 “I fear getting a serious disease” (2.75). It is interesting to note that the highest mean scores were on items related to diseases, especially among psychiatric patients. In the present study, the ASDA is measuring a trait because the high score of test-retest reliability refers to high temporal stability and corroborate the trait-like nature of scores. It is doubtable that the ASDA scores change much over adulthood except after trauma like getting cancer or losing a significant other.

The present results must be viewed within the limitations imposed by the data. Despite the good psychometric characteristics of the ASDA, the recruitment of the sample was not random, but rather one of convenience. Other multidimensional instruments such as the CLFDS, Multidimensional Fear of Death Scale (MFODS), Death Attitude Profile-Revised (DAP-R), and Multidimensional Orientation toward Dying and Death Inventory (MODDI-F) can be investigated in relation to the ASDA and its Farsi version in future studies. However, the ASDA may be useful in research on death anxiety among Iranian college students and psychiatric outpatients, and it is hoped that this study will stimulate further cross-cultural research on the ASDA.

## Figures and Tables

**Table 1:** Factor loadings of the Farsi version of the Arabic Scale of Death Anxiety (ASDA) in Iranian students (*N* = 252).

(ASDA) Items	Component			
	1	2	3	4
(1) I fear death whenever I become ill.	**.602**	.312	.098	.186
(2) I fear looking at the dead.	.143	**.821**	.230	.060
(3) I fear visiting graves.	.108	.237	**.712**	.014
(4)The possibility of having a surgical operation terrifies me.	**.551**	.287	.013	.072
(5) I am afraid of suffering heart attack.	**.787**	.179	.139	.042
(6) I worry that death deprives me of someone dear to me.	.312	.228	.034	.252
(7) I am apprehensive of unknown things after death.	.291	.275	.131	**.677**
(8) I am afraid of looking at a corpse.	.154	**.802**	.311	.124
(9) I fear the torture of the grave.	.088	.147	.259	**.761**
(10) I fear getting a serious disease.	**.814**	.053	.236	.120
(11) Witnessing the burial procedure terrifies me.	.143	.415	**.660**	.246
(12) I dread walking in graveyards.	.164	.175	**.809**	.163
(13) I am preoccupied with thinking about what will happen after death.	.200	.007	.025	**.757**
(14) I am afraid of sleeping and not waking up again.	**.606**	.137	.143	.303
(15) The pain accompanying death terrifies me.	**.590**	.208	−.182	.352
(16) I get upset by witnessing a funeral.	.223	**.784**	.270	.141
(17) The sight of a dying person frightens me.	.245	**.680**	.084	.175
(18) Talking about death upsets me.	.348	.406	.371	.144
(19) I am afraid of getting cancer.	**.859**	−.021	.253	.071
(20) I fear death.	**.612**	.331	.092	.353

Eigenvalue	4.33	3.35	2.26	2.25

% of variance	21.69	16.76	11.30	11.27
% of total variance	61.03			

Factor 1 (items: 1, 4, 5, 10, 14, 15, 19, and 20): fear of lethal disease and death fear; Factor 2 (items: 2, 8, 16, and 17): fear of dead people; Factor 3 (items: 3, 11, and 12): fear of tombs; Factor 4 (items: 7, 9, and 13): fear of postmortem events.

**Table 2:** Factor loadings of the Farsi version of the Arabic Scale of Death Anxiety (ASDA) in Iranian psychiatric outpatients (*N* = 52).

(ASDA) Items	Component			
	1	2	3	4
(1) I fear death whenever I become ill.	.247	.465	.341	.341
(2) I fear looking at the dead.	.095	**.796**	.257	−.187
(3) I fear visiting graves.	.112	.182	**.857**	.001
(4) The possibility of having a surgical operation terrifies me.	**.621**	.348	.179	−.025
(5) I am afraid of suffering heart attack.	**.833**	.207	.092	.242
(6) I worry that death deprives me of someone dear to me.	−.068	**.666**	−.059	.186
(7) I am apprehensive of unknown things after death.	.197	**.787**	.082	.179
(8) I am afraid of looking at a corpse.	.367	.495	**.548**	−.103
(9) I fear the torture of the grave.	.216	**.638**	−.038	.393
(10) I fear getting a serious disease.	**.803**	.026	.002	.364
(11) Witnessing the burial procedure terrifies me.	**.616**	.298	.479	.072
(12) I dread walking in graveyards.	.157	−.067	**.792**	.304
(13) I am preoccupied with thinking about what will happen after death.	.038	.283	.475	**.680**
(14) I am afraid of sleeping and not waking up again.	.291	.050	**.500**	.479
(15) The pain accompanying death terrifies me.	.493	.258	−.038	.495
(16) I get upset by witnessing a funeral.	**.658**	.495	.209	.238
(17) The sight of a dying person frightens me.	**.539**	**.580**	.306	−.006
(18) Talking about death upsets me.	.462	**.638**	.250	.263
(19) I am afraid of getting cancer.	**.748**	−.157	.302	−.090
(20) I fear death.	**.521**	.457	.444	.068

Eigenvalue	8.79	2.01	1.64	1.15

% of variance	43.97	10.05	8.21	5.75
% of total variance	67.99			

Factor 1 (items: 4, 5, 10, 11, 16, 17, 19, and 20): fear of lethal disease; Factor 2 (items: 2, 6, 7, 9, 17, and 18): fear of dead people and death fear; Factor 3 (items: 3, 8, 12, and 14): fear of tombs; Factor 4 (item 13): fear of postmortem events.

**Table 3:** Pearson correlations (*r*) between the Arabic Scale of Death Anxiety (ASDA) and other scales in Iranian college students.

Scales	*r* with ASDA
Death Obsession Scale	0.46^*∗*^
Death Depression Scale	0.56^*∗*^
Templer's Death Anxiety Scale	0.41^*∗*^
Wish to be Dead Scale	0.40^*∗*^
Love of Life Scale	−0.02

^*∗*^ *p* < 0.001 (two-tailed).

**Table 4:** Descriptive statistics of the Arabic Scale of Death Anxiety (ASDA) in college students from eight Arab, Western, and Iran countries.

Country	Men			Women			M. Diff.	*t*	*p*	*d*
	*N*	M	SD	*N*	M	SD				
Egypt	210	52.91	16.75	208	65.49	15.50	12.58	7.97	0.0001	.78^*∗∗*^
Kuwait	259	57.34	16.41	250	69.95	17.05	12.61	8.50	0.0001	.75^*∗∗*^
Lebanon	141	43.05	15.26	125	56.28	15.72	13.23	6.96	0.0001	.86^*∗∗∗*^
Syria	353	51.84	16.22	356	56.01	16.78	4.17	3.36	0.001	.25^*∗*^
Spain	134	41.57	13.28	525	51.29	16.05	9.72	7.24	0.0001	.70^*∗∗*^
United Kingdom	98	47.60	14.87	107	48.64	14.85	1.04	0.50	—	—
United States	87	35.84	14.22	145	45.17	14.85	9.33	4.76	0.0001	.64^*∗∗*^
Iran	128	42.82	13.99	124	39.94	11.15	2.87	1.80	0.001	.23^*∗*^

^*∗*^Small effect size; ^*∗∗*^medium effect size; ^*∗∗∗*^large effect size.
